# Effectiveness, Safety, and Costs of Thromboprophylaxis with Enoxaparin or Unfractionated Heparin Among Medical Inpatients With Chronic Obstructive Pulmonary Disease or Heart Failure

**DOI:** 10.36469/001c.92408

**Published:** 2024-02-20

**Authors:** Alpesh N. Amin, Alex Kartashov, Wilson Ngai, Kevin Steele, Ning Rosenthal

**Affiliations:** 1 Department of Medicine University of California, Irvine; 2 PINC AI™ Applied Sciences, Premier Inc., Charlotte, North Carolina, USA; 3 Sanofi, Bridgewater, New Jersey, USA

**Keywords:** thromboprophylaxis, chronic heart failure, chronic obstructive pulmonary disease, enoxaparin, heparin, medical inpatients, cost analysis, bleeding

## Abstract

**Background:** Chronic obstructive pulmonary disease (COPD) and heart failure (HF) are risk factors for venous thromboembolism (VTE). Enoxaparin and unfractionated heparin (UFH) help prevent hospital-associated VTE, but few studies have compared them in COPD or HF.

**Objectives:** To compare effectiveness, safety, and costs of enoxaparin vs UFH thromboprophylaxis in medical inpatients with COPD or HF.

**Methods:** This retrospective cohort study included adults with COPD or HF from the Premier PINC AI Healthcare Database. Included patients received prophylactic-dose enoxaparin or UFH during a >6-day index hospitalization (the first visit/admission that met selection criteria during the study period) between January 1, 2010, and September 30, 2016. Multivariable regression models assessed independent associations between exposures and outcomes. Hospital costs were adjusted to 2017 US dollars. Patients were followed 90 days postdischarge (readmission period).

**Results:** In the COPD cohort, 114 174 (69%) patients received enoxaparin and 51 011 (31%) received UFH. Among patients with COPD, enoxaparin recipients had 21%, 37%, and 10% lower odds of VTE, major bleeding, and in-hospital mortality during index admission, and 17% and 50% lower odds of major bleeding and heparin-induced thrombocytopenia (HIT) during the readmission period, compared with UFH recipients (all *P* <.006). In the HF cohort, 58 488 (58%) patients received enoxaparin and 42 726 (42%) received UFH. Enoxaparin recipients had 24% and 10% lower odds of major bleeding and in-hospital mortality during index admission, and 13%, 11%, and 51% lower odds of VTE, major bleeding, and HIT during readmission (all *P* <.04) compared with UFH recipients. Enoxaparin recipients also had significantly lower total hospital costs during index admission (mean reduction per patient: COPD, 1280;HF,2677) and readmission (COPD, 379;HF,1024). Among inpatients with COPD or HF, thromboprophylaxis with enoxaparin vs UFH was associated with significantly lower odds of bleeding, mortality, and HIT, and with lower hospital costs.

**Conclusions:** This study suggests that thromboprophylaxis with enoxaparin is associated with better outcomes and lower costs among medical inpatients with COPD or HF based on real-world evidence. Our findings underscore the importance of assessing clinical outcomes and side effects when evaluating cost-effectiveness.

## INTRODUCTION

Hospital-associated venous thromboembolism (HA-VTE) consists of deep venous thrombosis (DVT) or pulmonary embolism (PE) that occurs during hospitalization or within 90 days after discharge. HA-VTE comprises approximately half of VTE events in the United States and globally^1,2^ and is a leading preventable cause of hospital-associated morbidity and mortality.^2–4^ In large cohort studies, approximately 0.4% to 1.4% of medical inpatients developed HA-VTE, a 38- to 100-fold higher rate than in the general population.^5–8^ Treating acute VTE costs approximately $12 000 to $15 000 in the first year (US dollars), and managing subsequent complications is conservatively estimated to cost another $18 000 to $23 000.^9^

**Figure 1.**
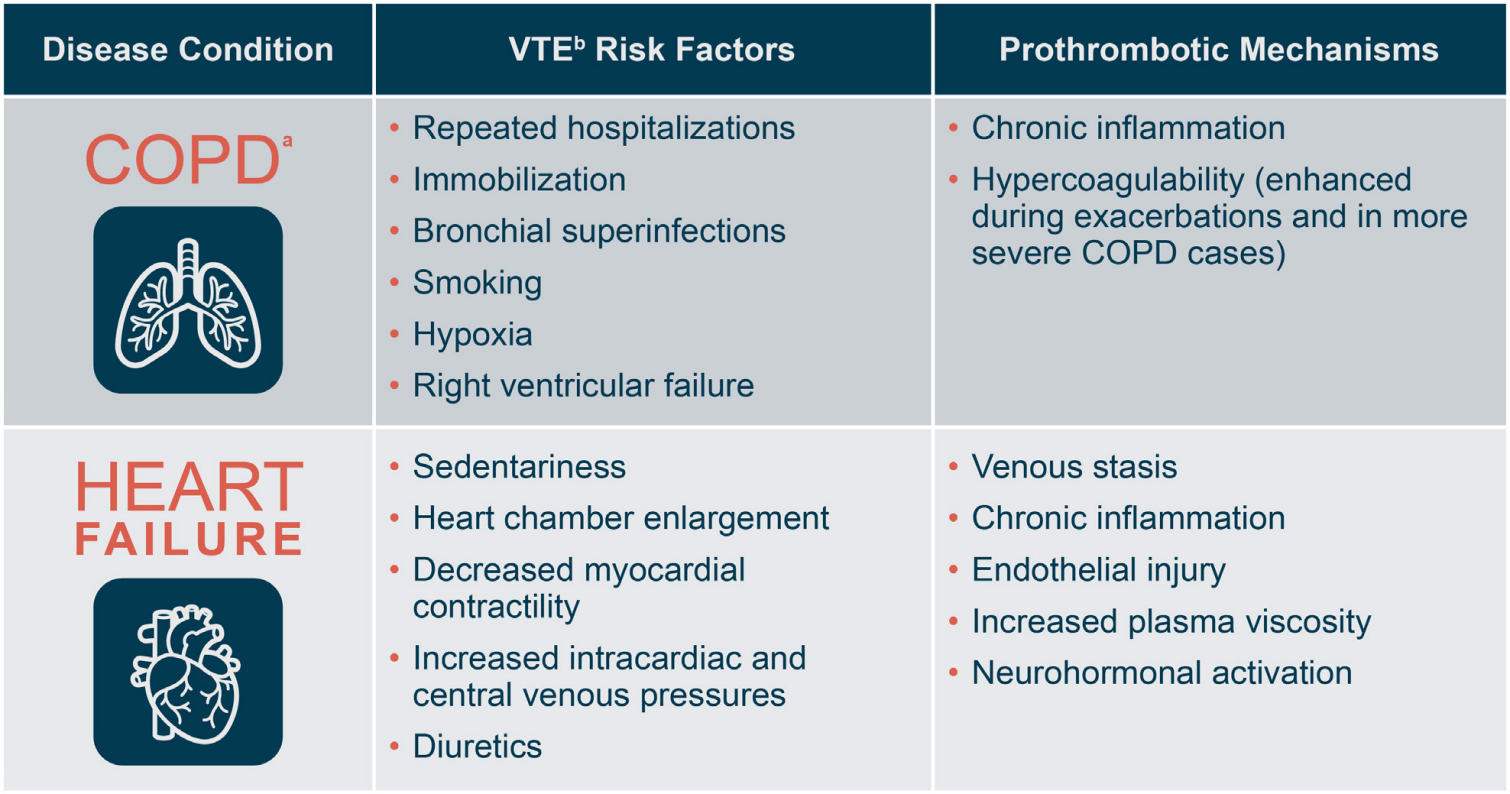
Risk Factors for Venous Thromboembolism and Prothrombotic Mechanisms in Obstructive Pulmonary Disease and Heart Failure COPD and heart failure are significant risk factors for venous thromboembolism, both in hospitalized patients and in the general population. This figure outlines the distinct risk factors and prothrombotic mechanisms that elevate VTE risk in each disease state.

Risk factors for VTE, including HA-VTE, include prolonged immobility, repeated hospitalizations, prothrombotic comorbidities (eg, malignancy, cardiovascular diseases, pulmonary diseases, infections, inflammatory bowel disease, obesity), mechanical ventilation, vascular injury, and personal or family history of thromboses.^10–14^ Among medically ill inpatients, chronic obstructive pulmonary disease (COPD) and heart failure (HF) are important risk factors for VTE. **Figure 1** shows contributing factors and prothrombotic mechanisms in each of these two disease states.^15–20^ In a large population-based study, adults with stage III/IV COPD had approximately double the risk of a VTE event compared with adults with normal airflow.^15^ In another large prospective study, 7.3% of patients admitted with COPD exacerbations were diagnosed with VTE within 48 hours.^21^ Among inpatients with HF, the incidence of symptomatic HA-VTE was 2.48% in a large meta-analysis,^18^ while new-onset HF was associated with a 2.2-fold increase in 30-day odds of VTE in a matched cohort study of inpatient Medicare claims data.^22^ Of note, HA-VTE significantly increases risk for mortality in both COPD and HF.^15,22–30^

For medical inpatients at risk for HA-VTE, pharmacologic VTE prophylaxis is cost-effective and generally well tolerated, according to the findings of randomized controlled trials, prospective observational studies, and meta-analyses.^31–36^ Numerous professional societies and healthcare quality and accreditation organizations recommend primary thromboprophylaxis for medically ill inpatients.^2,3,27,37–40^ Moreover, joint guidelines from the American Heart Association, American College of Cardiology, and Heart Failure Society of America specifically recommend VTE prophylaxis for patients hospitalized with HF.^41^ In the meta-analysis of inpatients with HF, the pooled incidence of symptomatic HA-VTE was 1.47% among patients who received thromboprophylaxis vs 3.73% among those who did not.^18^ Although data on VTE prophylaxis for patients with COPD is less extensive, the Global Initiative for Chronic Obstructive Lung Disease (GOLD) has recommended thromboprophylaxis for patients hospitalized with COPD exacerbations.^42^

Unfractionated heparin (UFH) and low-molecular-weight heparins (LMWHs), such as enoxaparin, are the most commonly used agents for pharmacologic thromboprophylaxis in hospitalized individuals.^13^ Although these agents have distinct mechanisms of actions, pharmacokinetics, and risk profiles, little evidence is available to help guide clinicians’ decisions regarding which one to use for thromboprophylaxis in the setting of COPD or HF. To help fill this gap, we compared real-world efficacy, safety, and economic outcomes among US adults who were medical inpatients, had a primary or secondary diagnosis of COPD or HF, and received thromboprophylaxis with UFH or enoxaparin during their hospital stay.

## METHODS

### Study Design and Data Source

This was a retrospective study of the Premier PINC AI™ Healthcare Database (PHD; formerly known as the Premier Healthcare Database), a service-level, all-payer hospital discharge database that covers approximately 25% of annual admissions in the US, including admissions from more than 850 rural and urban nongovernmental, nonprofit, community and teaching hospitals and health systems in 45 states and the District of Columbia. Each patient in the PHD is allocated a unique masked identifier that links visits within a hospital system. Data are extracted from standard hospital discharge files and include patient demographics, diagnoses at admission and discharge, comorbidities, and date-stamped, billed items linked to procedures, medical services, medications, laboratory tests, diagnostic and therapeutic services, and health status. Medication data are available for each day of the hospital stay and include medication type, dose, quantity, and cost. All data are de-identified, compliant with the Health Insurance Portability and Accountability Act (HIPAA), and exempt from institutional review board oversight as per 45 CFR §46.101(b)(4).

### Patients

Patients in this study were at least 18 years old, had a primary or secondary discharge diagnosis of COPD or HF based on concurrent *International Classification of Diseases* (ICD), *Ninth Revision, Clinical Modification* (CM) or *ICD Tenth Revision* CM codes (**Supplemental Material**), were hospitalized for 6 or more days, received 1 or more prophylactic dose of enoxaparin (≤40 mg/day) or UFH (≤15 000 IU/day) during their hospital stay, and were discharged between January 1, 2010, and September 30, 2016. For each patient, the index hospitalization was defined as the first admission that met these criteria. Because HA-VTE often is not identified until after discharge,^5^ admissions to the same hospital system within 90 days after index discharge were also identified and evaluated separately.

Patients were excluded from the study if they received enoxaparin, UFH, fondaparinux, dalteparin, or rivaroxaban during the 90 days prior to their index admission; if they had a diagnosis of VTE during the 90 days prior to or the first 2 days of the index admission; or if they received therapeutic-dose anticoagulants during the first 2 days of the index admission. Patients also were excluded if they had a diagnosis of a hemorrhagic disorder, thrombophilic condition, or active peptic ulcer; received dabigatran, warfarin, edoxaban, apixaban, rivaroxaban, or any combination of anticoagulants (of any type or class); or received mechanical VTE prophylaxis or underwent surgery or obstetric procedures during index admission or the 90 days beforehand. Patients with missing cost data also were excluded.

### Outcome Measures

The main clinical outcome measure was an event of VTE during index hospitalization or 90-day readmission period. Secondary clinical outcomes were in-hospital mortality, PE-related mortality, major bleeding, and heparin-induced thrombocytopenia (HIT) during index hospitalization or readmission period. Relevant ICD-9/10-CM diagnosis codes are provided in the **Supplemental Material**. Economic outcomes were the cost of pharmacologic thromboprophylaxis during index hospitalization (ie, the combined cost of all doses of UFH or enoxaparin), and total hospital costs during index hospitalization and readmission period. Costs were calculated per patient based on hospital chargemaster data and adjusted to 2017 US dollars based on the Consumer Price Index for all urban consumers for hospital and related services.

We assessed variables that might confound relationships between exposure (enoxaparin vs UFH) and study outcomes, including patient demographics (age, sex, race, payer type), visit characteristics (admission type and source, discharge disposition, intensive care unit [ICU] admission), comorbidities based on the Deyo modification of the Charlson Comorbidity Index (which adapts the CCI for use with administrative databases of ICD codes),^43^ and severity of illness based on the 3M All Patient Refined™ Diagnosis-Related Group (APR-DRG) Severity of Illness (SOI) score. The APR-DRG SOI is categorized as minor, moderate, major, or extreme and incorporates age, procedures, diagnoses at admission, and any additional diagnoses made during the hospital stay.^44^ We also evaluated individual comorbidities by analyzing ICD-9/10 diagnosis codes for myocardial infarction, lower limb fracture, inflammatory bowel disease, malignant hypertension (including renal disease with or without renal failure), nephrotic syndrome, and obesity (see **Supplemental Material**). These covariates were assessed because they are risk factors for the clinical outcomes of interest and may be associated with the exposure of interest (enoxaparin vs UFH). In addition, we evaluated the prevalence of COPD in the HF cohort, the prevalence of HF in the COPD cohort, and intubation (a risk factor for VTE) in both cohorts. Finally, we assessed hospital characteristics, including geographic region, population served (urban or rural), teaching status (teaching or non-teaching), and categorical bed number.

### Statistical Analyses

Analyses were performed using SAS v. 9.4 (SAS Institute Inc.). When *P* values were calculated, statistical significance was defined as *P* < .05. Patients were grouped according to whether they had received thromboprophylactic-dose UFH or enoxaparin during index hospitalization. Descriptive analyses of demographic data and visit, clinical, and hospital characteristics were reported as proportions and frequencies for categorical variables and means ± SD for continuous variables. To evaluate differences between groups, the χ^2^ test was used for categorical variables and Student’s *t*-test or the Wilcoxon rank sum test was used for continuous variables. To determine which test to use, normality of data was evaluated by the Kolmogorov-Smirnov test and by histogram.

Multivariable logistic regression models were created to compare the adjusted odds of VTE, in-hospital mortality, PE-related mortality, and major bleeding between the enoxaparin and UFH groups for both the index hospitalization period and the 90-day readmission period. To control for possible confounding, models were adjusted for patients’ demographic characteristics, visit and hospital characteristics, severity of illness (APR-DRG SOI score and ICU stay), CCI category, intubation, and individual comorbidities. We did not adjust for serum creatinine concentration because this is not included in the PHD.

For each group, we also calculated the unadjusted mean (± SD) cost of thromboprophylaxis per patient during index hospitalization, and the unadjusted mean (± SD) total cost of hospitalization per patient during index hospitalization and 90-day readmission period. To minimize the effect of outliers, costs were winsorized at the 2.5th percentile and the 99th percentile (ie, values <2.5th percentile were assigned the value of the 2.5th percentile, and values above the 99th percentile were assigned the value of the 99th percentile). Generalized linear models with gamma link function were constructed, and results were reported as adjusted means and CIs. Each regression model was evaluated for fitness and convergence of algorithms. Regression diagnostics of multicollinearity between variables showed no need to delete any variable.

## RESULTS

### COPD Cohort

Among 288 869 patients with COPD, 165 185 met inclusion criteria, of whom 114 174 (69%) received thromboprophylactic-dose enoxaparin and 51 011 (31%) received thromboprophylactic-dose UFH during index hospitalization. **Table 1** compares the two exposure groups based on demographic, clinical, and hospital characteristics. On average, UFH recipients were 2 years older and were significantly more likely to be male, non-white, and transferred from an acute care facility compared with patients who received enoxaparin (all *P* < .0001). Patients who received enoxaparin had a 0.4-day shorter mean hospital length of stay (LOS) and a significantly lower rate of ICU admission (27% vs 34%, *P* < .0001). They also had a lower comorbidity burden, with a mean CCI score of 2.7 ± 2.1 compared with 3.4 ± 2.3 in the UFH group (*P* < .0001). The largest differences in prevalence of individual comorbidities were for renal disease (11% in the enoxaparin group vs 24% in the UFH group) and HF (27% vs 37%, respectively). Malignant hypertension was the only comorbidity that was more prevalent in the enoxaparin group vs the UFH group (10% vs 9%, *P* < .0001).

**Table 1.** Demographic, Clinical, and Hospital Characteristics of Medically Ill US Adults With COPD Who Received Thromboprophylaxis With Enoxaparin or Unfractionated Heparin During Their Index Hospitalization

**Characteristics**	**Enoxaparin (N = 114 174)**	**Unfractionated Heparin (N = 51 011)**	***P* Value**
Demographic characteristics			
Age, y (mean ± SD)	66 ± 14	68 ± 14	<.0001
Female sex	67 525 (59)	27 506 (54)	<.0001
Race			<.0001
White	89 104 (78)	36 368 (71)	
Black	12 009 (11)	6458 (13)	
Other	12 724 (11)	8066 (16)	
Unknown	337 (0.3)	119 (0.3)	
Payer type			<.0001
Private	18 870 (17)	8021 (16)	
Medicaid	13 251 (12)	5745 (11)	
Medicare	74 783 (66)	35 181 (69)	
Uninsured	5761 (5.1)	1673 (3.3)	
Unknown	1509 (1.3)	391 (0.8)	
Visit characteristics			
Admission source			<.0001
Home	91 575 (80)	38 730 (76)	
Transfer from acute care facility	8527 (8)	5911 (12)	
Transfer from skilled nursing facility	2343 (2)	1451 (3)	
Emergency room	7613 (7)	3061 (6)	
Other/unknown	4116 (4)	1858 (3.6)	
Admission type			<.0001
Emergency	86 911 (76)	37 657 (74)	
Urgent	17 554 (15)	7704 (15)	
Elective	8895 (8)	5201 (10)	
Trauma	273 (0.2)	202 (0.4)	
Unknown	541 (0.5)	247 (0.5)	
Discharge status			<.0001
Died	3820 (3)	2620 (5)	
Home	83 740 (73)	34 438 (68)	
Transferred to acute care setting	1585 (1)	855 (2)	
Transferred to nursing or rehabilitation facility	23 476 (21)	12 320 (24)	
Other	1553 (1.4)	778 (2)	
ICU admission stay	31 006 (27)	17 164 (34)	<.0001
Hospital length of stay (days) (mean + SD)	8.5 ± 4.1	8.9 ± 5.2	<.0001
Clinical characteristics			
Severity of illness (APR-SOI)			<.0001
Minor	4989 (4)	1649 (3)	
Moderate	32 253 (28)	11 537 (23)	
Major	55 615 (49)	24 555 (48)	
Extreme	21 317 (19)	13 270 (26)	
CCI score^a^			<.0001
0	0 (0)	0 (0)	
1-2	67 677 (59)	22 144 (43)	
>3	46 497 (41)	28 867 (57)	
Myocardial infarction	10 044 (9)	6196 (12)	<.0001
Heart failure	31 391 (27)	19 124 (37)	<.0001
Peripheral vascular disease	8001 (7)	4876 (10)	<.0001
Cerebrovascular disease	6952 (6)	4635 (9)	<.0001
Dementia	7242 (6)	3944 (7)	<.0001
Rheumatologic disease	4245 (4)	1815 (4)	.1101
Peptic ulcer disease	864 (0.8)	486 (1)	<.0001
Mild liver disease	1106 (1)	704 (1)	<.0001
Diabetes	30 090 (26)	14 402 (28)	<.0001
Diabetes with chronic complications	5671 (5)	3670 (7)	<.0001
Hemiplegia or paraplegia	1592 (1)	893 (2)	<.0001
Renal disease	12 431 (11)	12 088 (24)	<.0001
Any malignancy, including leukemia and lymphoma	11 388 (10)	6251 (12)	<.0001
Moderate or severe liver disease	330 (0.3)	280 (0.6)	<.0001
Metastatic solid tumor	4776 (4)	2501 (5)	<.0001
AIDS/HIV	348 (0.3)	382 (0.8)	<.0001
Inflammatory bowel disease	735 (0.6)	329 (0.6)	.9774
Fracture of lower limb	247 (0.2)	94 (0.2)	.1847
Nephrotic syndrome	65 (0.1)	73 (0.1)	<.0001
Intubation	11 643 (10)	6769 (13)	<.0001
Malignant hypertension	11 212 (10)	4342 (8)	<.0001
Obesity	24 213 (21)	10 639 (21)	.1064
HIV infection	652 (0.6)	601 (1)	<.0001
Hospital characteristics			
Geographic region			<.0001
Northeast	15 977 (14)	17 573 (34)	
Midwest	21 019 (18)	10 340 (20)	
South	62 495 (55)	17 013 (33)	
West	14 683 (13)	6085 (12)	
Bed size			<.0001
1-299	44 653 (39)	17 265 (34)	
300-499	37 189 (33)	17 461 (34)	
>500	32 332 (28)	16 285 (32)	
Population served			<.0001
Rural	16 551 (15)	4470 (8.8)	
Urban	97 623 (86)	46 541 (91)	
Teaching status			<.0001
Non-teaching	74 830 (66)	23 037 (45)	
Teaching	39 334 (34)	27 974 (55)	

Data are presented as n (%) unless otherwise indicated.

Abbreviations: APR SOI, All Patient Refined^TM^ Diagnosis-Related Group Severity of Illness score at discharge; CCI, Charlson Comorbidity Index; COPD, chronic obstructive pulmonary disease; ICU, intensive care unit.

During index hospitalization, the enoxaparin group had significantly lower unadjusted rates of VTE (0.34% vs 0.59% of UFH recipients), overall mortality (3.4% vs 5.1%), PE-related mortality (0.03% vs 0.07%), and major bleeding (2% vs 3%) (all *P* < .0001), and a significantly lower percentage of APR-SOI scores in the extreme category (19% vs 26% in the UFH group, *P* < .0001), indicating a lower likelihood of severe illness at index discharge. No events of HIT were reported in either exposure group during index hospitalization.

The risk of readmissions during the 90-day readmission period was similar between the enoxaparin and UFH groups (46% vs 45%, *P* = .06). Prior to multivariable adjustment, the enoxaparin group had significantly lower rates of VTE (2.5% vs 2.9% in the UFH group, *P* = .0009), major bleeding (2.9% vs 3.8%, *P* < .0001), HIT (0.06% vs 0.14%, *P* <.001), and overall in-hospital mortality (4.6% vs 5.3%, *P* < .0001) during the readmission period. Unadjusted rates of PE-related mortality during the readmission period were low and comparable between groups (0.14% and 0.20%, *P* = .0588).

**Table 2** shows multivariable analyses comparing clinical outcomes between the enoxaparin and UFH groups during the index hospitalization period and the 90-day readmission period. The enoxaparin group had significantly lower adjusted odds of VTE (adjusted odds ratio [aOR] = 0.79, 95% CI: 0.67-0.93, *P* = .0059), in-hospital mortality (aOR = 0.90, 95% CI: 0.85-0.96, *P* = .0008), and major bleeding (aOR = 0.63, 95% CI: 0.58-0.68, *P* < .0001) during index hospitalization and significantly lower adjusted odds of major bleeding (aOR 0.83, 95% C, 0.76-0.91; *P =* .0001) and HIT (aOR = 0.50, 95% CI, 0.29-0.85, *P* < .0001) during the readmission period.

**Table 2.** Multivariable Analysis of Clinical Outcomes Among Medically Ill US Adults With COPD Who Received Thromboprophylaxis With Enoxaparin or Unfractionated Heparin During Their Index Hospitalization

	**Enoxaparin**	**Unfractionated Heparin**	**Enoxaparin (vs Unfractionated Heparin)**	
			**Adjusted OR^b^ (95% CI)**	***P* Value**
Index hospitalization period	N = 114 174	N = 51 011		
VTE event	385 (0.34)	299 (0.59)	0.79 (0.67-0.93)	0.0059
In-hospital mortality	3820 (3.35)	2620 (5.1)	0.90 (0.85-0.96)	0.0008
PE-related mortality	36 (0.03)	38 (0.07)	0.81 (0.49-1.34)	0.4085
Major bleeding	1978 (1.73)	1621 (3.18)	0.63 (0.58-0.68)	<0.0001
90-day readmission period	n = 51 945 (46%)	n = 22 952 (45%)		
VTE event	1272 (2.45)	658 (2.87)	0.94 (0.85-1.04)	0.2395
In-hospital mortality	2365 (4.55)	1206 (5.25)	1.08 (1.00-1.17)	0.0465
PE-related mortality	75 (0.14)	47 (0.20)	0.81 (0.54-1.21)	0.2967
Major bleeding	1504 (2.90)	879 (3.83)	0.83 (0.76-0.91)	0.0001
HIT^a^	30 (0.06)	33 (0.14)	0.50 (0.29 − 0.85)	<0.001

Data are presented as n (%) unless otherwise indicated.

^a^No events of HIT were coded for the index admission period.

^b^Adjusted for age, sex, race, payer type, admission source, admission type, ICU stay (yes/no), geographic area, obesity, severity of illness, CCI category, myocardial infarction, inflammatory bowel disease, obesity, nephrotic syndrome, fracture of lower limb, heart failure, intubation, malignant hypertension, and hospital characteristics (teaching vs non-teaching, hospital beds)

Abbreviations: CI, confidence interval; COPD, chronic obstructive pulmonary disease; HIT, heparin-induced thrombocytopenia; OR, odds ratio; PE, pulmonary embolism; VTE, venous thromboembolism.

### Heart Failure Cohort

Among 199 022 patients with HF, 101 214 met study inclusion criteria, of whom 58 488 (58%) received thromboprophylactic-dose enoxaparin and 42 726 (42%) received thromboprophylactic-dose UFH during index hospitalization. **Table 3** compares the two exposure groups. Mean age was 72 ± 14 years in each group. Similar to the COPD cohort, recipients of UFH were significantly more likely to be male, non-White, and transferred from an acute care facility (all *P* < .0001), while patients who received enoxaparin had a 0.4-day shorter mean LOS (*P* < .0001) and a lower rate of ICU admissions during index hospitalization (31% vs 35% in the UFH group, *P* < .0001). Enoxaparin recipients had a lower overall comorbidity burden vs the UFH group (mean CCI scores, 3.4 ± 1.9 vs 3.7 ± 2.0) but a higher prevalence of malignant hypertension (12% vs 11%, respectively) and obesity (25% vs 23%, respectively) (both *P* < .0001). Among all the individual comorbidities that were evaluated, the greatest differences in prevalence between exposure groups were for renal disease (41% of UFH recipients vs 25% of enoxaparin recipients) and COPD (45% vs 54%, respectively).

**Table 3.** Demographic, Clinical, and Hospital Characteristics Among Medically Ill US Adults With Heart Failure Who Received Thromboprophylaxis With Enoxaparin or Unfractionated Heparin During Their Index Hospitalization

**Characteristics**	**Enoxaparin** (N = 58 488)	**Unfractionated Heparin** (N = 42 726)	***P* Value**
Demographic characteristics			
Age, y (mean ± SD)	72 ± 14	72 ± 14	.2583
Female sex	31 754 (54)	20 858 (49)	<.0001
Race			<.0001
White	43 305 (74)	29 416 (69)	
Black	7826 (13)	6541 (15)	
Other	7130 (12)	6670 (16)	
Unknown	227 (0.4)	99 (0.2)	
Payer type			<.0001
Private	6611 (11)	5488 (13)	
Medicaid	4693 (8)	3318 (8)	
Medicare	44 327 (76)	32 330 (76)	
Uninsured	2192 (4)	1251 (3)	
Unknown	665 (1)	339 (0.8)	
Visit characteristics			
Admission source			<.0001
Home	45 161 (77)	31 506 (74)	
Transfer from acute care facility	5226 (9)	5610 (13)	
Transfer from skilled nursing facility	1885 (3)	1549 (4)	
Emergency room	4279 (7)	2613 (6)	
Other/unknown	1937 (3)	1448 (3)	
Admission type			<.0001
Emergency	45 044 (77)	31 707 (74)	
Urgent	9309 (16)	7271 (17)	
Elective	3705 (6)	3301 (8)	
Trauma	157 (0.3)	226 (0.5)	
Unknown	273 (0.5)	221 (0.5)	
Discharge status			<.0001
Expired	3035 (5)	2865 (7)	
Home	35 773 (61)	25 940 (61)	
Transferred to another acute care setting	1156 (2)	974 (3)	
Transferred to nursing or rehabilitation facility	17 820 (30)	12 397 (29)	
Other	704 (1)	550 (1)	
ICU admission	18 058 (31)	14 824 (35)	<.0001
Hospital length of stay (days) (mean ± SD)	8.8 ± 5.0	9.2 ± 5.4	<.0001
Clinical characteristics			
Severity of illness (APR-SOI)			<.0001
Minor	715 (1)	604 (1)	
Major	9404 (16)	5304 (12)	
Moderate	32 009 (55)	22 869 (54)	
Extreme	16 360 (28)	13 949 (33)	
CCI score^a^			<.0001
0	0 (0)	0 (0)	
1-2	21 694 (37)	12 796 (30)	
>3	36 794 (63)	29 930 (70)	
Myocardial infarction	9857 (17)	8509 (20)	<.0001
Peripheral vascular disease	5509 (9)	4608 (11)	<.0001
Cerebrovascular disease	5919 (10)	4828 (11)	<.0001
Dementia	6757 (12)	4617 (11)	.0002
COPD	31 391 (54)	19 124 (45)	<.0001
Rheumatologic disease	2054 (4)	1282 (3)	<.0001
Peptic ulcer disease	457 (0.8)	395 (0.9)	.0138
Mild liver disease	680 (1)	677 (2)	<.0001
Diabetes	19 962 (34)	14 490 (34)	.4732
Diabetes with chronic complications	5052 (9)	4985 (12)	<.0001
Hemiplegia or paraplegia	1242 (2)	899 (2)	.8322
Renal disease	14 454 (25)	17 555 (41)	<.0001
Any malignancy, including leukemia and lymphoma	3376 (6)	2272 (5)	.0019
Moderate or severe liver disease	255 (0.4)	314 (0.7)	<.0001
Metastatic solid tumor	1236 (2)	822 (2)	.0350
AIDS/HIV	63 0.1)	119 (0.3)	<.0001
Inflammatory bowel disease	272 (0.5)	195 (0.5)	.8409
Fracture of lower limb	175 (0.3)	75 (0.2)	<.0001
Nephrotic syndrome	111 (0.2)	146 (0.3)	<.0001
Intubation	6472 (11)	5505 (13)	<.0001
Malignant hypertension	7260 (12)	4846 (11)	<.0001
Obesity	14 466 (25)	9630 (23)	<.0001
HIV infection	143 (0.2)	213 (0.5)	<.0001
Hospital characteristics			
Geographic region			<.0001
Northeast	8745 (15)	14 404 (34)	
Midwest	11 065 (19)	8297 (19)	
South	31 677 (54)	15 089 (35)	
West	7001 (12)	4936 (12)	
Bed size			<.0001
1-299	21 112 (36)	12 959 (30)	
300-499	19 172 (33)	14 652 (34)	
>500	18 204 (31)	15 115 (35)	
Population served			<.0001
Rural	7978 (14)	3530 (8)	
Urban	50 510 (86)	39 196 (92)	
Teaching status			<.0001
Non-teaching	36 097 (62)	19 215 (45)	
Teaching	22 391 (38)	23 511 (55)	

Data are presented as n (%) unless otherwise indicated.

^a^Myocardial infarction, heart failure, peripheral vascular disease, history of cerebrovascular accident and transient ischemic attacks, dementia, COPD, connective tissue disease, mild or moderate to severe liver disease, diabetes mellitus uncomplicated or with end-organ damage, hemiplegia, mild or moderate to severe renal disease, malignancy, and HIV-positive status.

Abbreviations: APR SOI, All Patient Refined™ Diagnosis-Related Group Severity of Illness score at discharge; CCI, Charlson Comorbidity Index; COPD chronic obstructive pulmonary disease, ICU intensive care unit.

During the index hospitalization period, unadjusted rates of VTE were 0.50% in the enoxaparin group and 0.66% in the UFH group (*P* < .0007). The enoxaparin group had significantly lower unadjusted rates of in-hospital mortality (5.2% vs 6.7%, *P* < .0001), PE-related mortality (0.05% vs 0.09%, *P* = .03), and major bleeding (1.9% vs 3.0%, *P* < .0001) and a significantly lower rate of extreme APR-SOI scores at discharge (28% vs 33%, *P* < .0001). Events of HIT were not reported in either group during index hospitalization.

The risk of 90-day readmissions was 44% in each exposure group. Among readmitted patients, those who had received enoxaparin had significantly lower unadjusted rates of major bleeding (3.4% vs 4.0% in the UFH group) and HIT (0.07% vs 0.16% in the UFH group) (both *P* <.01). The two exposure groups did not significantly differ based on unadjusted rates of VTE (2.3% and 2.5%, respectively), overall mortality (6.4% and 6.5%), and PE-related mortality (0.13% and 0.17%).

In the multivariable analysis (**Table 4**), enoxaparin vs UFH was associated with significantly lower adjusted odds of in-hospital mortality (aOR = 0.90, 95% CI: 0.85-0.96, *P* = .0007) and major bleeding (aOR = 0.76; 95% CI: 0.69-0.83, *P* < .0001) during index hospitalization. Patients who received enoxaparin during index hospitalization also had significantly lower adjusted odds of major bleeding, VTE, and HIT during the 90-day readmission period (adjusted ORs, 0.89 [95% CI: 0.80-0.99, *P* = .0001], 0.87 [95% CI: 0.76-0.99, *P* = .0389], and 0.49 [95% CI: 0.27-0.88, *P* <.001], respectively).

**Table 4.** Multivariable Analysis of Clinical Outcomes Among Medically Ill US Adults With Heart Failure Who Received Thromboprophylaxis With Enoxaparin or Unfractionated Heparin During Their Index Hospitalization

	**Enoxaparin**	**Unfractionated Heparin**	**Enoxaparin (vs Unfractionated Heparin)**	
			**Adjusted OR^b^ (95% CI)**	***P* Value**
Index hospitalization period	N = 58 488	N = 42 726		
VTE event	294 (0.50)	284 (0.66)	0.88 (0.73-1.05)	.1548
In-hospital mortality	3035 (5.19)	2865 (6.71)	0.90 (0.85-0.96)	.0007
PE-related mortality	32 (0.05)	39 (0.09)	0.87 (0.52-1.45)	.5975
Major bleeding	1096 (1.87)	1268 (2.97)	0.76 (0.69-0.83)	<.0001
90-day readmission period	n = 25 921 (44%)	n = 18 768 (44%)		
VTE event	598 (2.31)	472 (2.51)	0.87 (0.76-0.99)	.0389
In-hospital mortality	1657 (6.39)	1223 (6.52)	1.08 (1.00-1.18)	.06
PE-related mortality	34 (0.13)	31 (0.17)	0.78 (0.45-1.35)	.3676
Major bleeding	884 (3.41)	750 (4.00)	0.89 (0.80-0.99)	.0001
HIT^a^	20 (0.08)	35 (0.19)	0.49 (0.27-0.88)	<.001

Data are presented as n (%) unless otherwise indicated.

^a^No events of HIT were coded for the index admission period

^b^Adjusted for age, sex, race, payer type, admission source and type, intensive care unit stay (yes vs no), geographic area, obesity, severity of illness, Charlson Comorbidity Index category, chronic obstructive pulmonary disease, myocardial infarction, inflammatory bowel disease, obesity, nephrotic syndrome, fracture of lower limb, intubation, malignant hypertension, and hospital characteristics (teaching vs non-teaching, hospital bed size category, urban vs rural).

Abbreviations: CI, confidence interval; HIT, heparin-induced thrombocytopenia; OR, odds ratio; PE, pulmonary embolism; VTE venous thromboembolism.

### Economic Outcomes

**Table 5** shows economic outcomes for the COPD cohort and the HF cohort. In the COPD cohort, the adjusted mean cost of thromboprophylaxis per patient was US$77 higher in the enoxaparin group than the UFH group (*P* < .0001). However, total hospital costs were significantly lower in the enoxaparin group during the index hospitalization (mean reduction per patient: $1280, *P* < .0001) and the 90-day readmission period (mean reduction per patient: $379, *P* < 0001). In the HF cohort, the adjusted mean cost of thromboprophylaxis per patient was $70 higher in the enoxaparin group than the UFH group (*P* < .0001), but once again, total hospital costs were significantly lower in the enoxaparin group during the index hospitalization (mean reduction per patient: $2677, *P* < .0001) and the 90-day readmission period (mean reduction per patient: $1024, *P* < .0001).

**Table 5.** Economic Outcomes Among Medically Ill US Adults With COPD or HF Who Received Thromboprophylaxis With Enoxaparin or Unfractionated Heparin During Their Index Hospitalization

	**COPD Cohort**		
	**Adjusted Mean Estimates^a^ (95% CI)**		
	**Unfractionated Heparin**	**Enoxaparin**	**P Value**
Index hospitalization period			
Total hospital costs	16 976(16 601-$17 360)	15 696(15 349-$16 051)	<.0001
Cost of pharmacologic prophylaxis	68.60(65.70-$71.62)	145.83(139.67-$152.26)	<.0001
90-day readmission period			
Total hospital costs	5932(5302-$6636)	5552(4963-$6212)	<.0001
	**HF Cohort**		
	**Adjusted Mean Estimates^b^ (95% CI)**		
	**Unfractionated Heparin**	**Enoxaparin**	**P Value**
Index hospitalization period			
Total hospital costs	25 752(24 665-$26 887)	23 075(22 101-$24 092)	<.0001
Cost of pharmacologic prophylaxis	54.37(49.93-$59.21)	124.38(114.22-$135.44)	<.0001
90-day readmission period			
Total hospital costs	10 750(8824-$13 097)	9726(7984-$11 849)	<.0001

Note: Costs are per patient. Data are presented in 2017 US dollars as mean ± SD or mean (95% CI) unless otherwise indicated.

^a^Adjusted for patient characteristics (age, sex, race, payer), visit characteristics (admission source and type, and ICU admission), clinical characteristics (severity of illness, CCI score, HF, myocardial infarction, inflammatory bowel disease, nephrotic syndrome, fracture of lower limb, intubation, malignant hypertension), and hospital characteristics (teaching status, bed number category, geographic region, and rurality)

^b^Adjusted for patient characteristics (age, sex, race, payer), visit characteristics (admission source and type, and ICU admission), clinical characteristics (severity of illness, CCI score, COPD, myocardial infarction, inflammatory bowel disease, nephrotic syndrome, fracture of lower limb, intubation, malignant hypertension), and hospital characteristics (teaching status, bed number category, geographic region, and rurality).

Abbreviations: CI, confidence interval; COPD, chronic obstructive pulmonary disease; HF, heart failure.

## DISCUSSION

In this large, real-world observational study, we compared clinical and economic outcomes among inpatients with COPD or HF who received thromboprophylaxis with enoxaparin or UFH. During the index hospitalization period, thromboprophylaxis with enoxaparin was associated with significantly lower adjusted odds of VTE, major bleeding, and in-hospital mortality among patients with COPD and with significantly lower adjusted odds of major bleeding and in-hospital mortality among patients with HF. During the 90 days after index discharge (the readmission period), enoxaparin was associated with significantly lower adjusted odds of bleeding and HIT in both cohorts, and with significantly lower adjusted odds of VTE in patients with HF. Enoxaparin cost more than UFH, but in both the COPD and HF cohorts, enoxaparin recipients had significantly lower adjusted mean hospital costs both during index hospitalization and if they were readmitted.

LMWHs such as enoxaparin comprise a distinct drug class that differs from UFH in several important ways. LMWHs have more predictable bioavailability and pharmacokinetics, which facilitates fixed-dose prophylaxis without the need for laboratory monitoring. They also have a longer anticoagulant effect, which permits once- or twice-daily dosing but makes it more challenging to rapidly halt anticoagulation when needed (although protamine sulfate can be used as a reversal agent, it only partially inactivates LMWH activity).^45–54^ In contrast, UFH has a rapid onset of action and undergoes efficient metabolic clearance, making it easier to titrate doses and stop anticoagulation rapidly (protamine sulfate efficiently reverses UFH activity).^55^ Because UFH does not undergo significant renal clearance, it is the thromboprophylactic agent of choice for patients with renal insufficiency or renal failure.^54^ However, the unpredictable pharmacokinetics of UFH means that patients require regular laboratory monitoring.^56–58^

In the majority of published randomized controlled trials, observational studies, decision analyses, and prospective economic evaluations of medical inpatients, pharmacologic thromboprophylaxis with LMWH agents was at least as effective as UFH for the prevention of HA-VTE and also was associated with lower rates of adverse events and significant reductions in total hospital costs.^51,59–63^ Many of these studies included patients with COPD or HF, but almost none focused on these patients, and subgroup data usually were not reported. An exception is a multicenter, randomized, open-label study comparing thromboprophylaxis with enoxaparin or UFH in 451 patients with severe respiratory disease or HF.^46^ In unadjusted analyses, patients with HF who received enoxaparin had 60% to 64% lower rates of DVT and PE compared with their counterparts who received UFH. A somewhat less pronounced effect was observed in patients with severe respiratory disease. The study did not report cost data.

As in this prior study, we found that thromboprophylaxis with enoxaparin vs UFH was associated with a significant reduction in rates of VTE, although differences did not always retain statistical significance in multivariable analyses. Rates of VTE during index hospitalization were less than 1% in all exposure groups in our study, which resembles findings from recent cohort studies of medical inpatients.^5,6^ The overall rate of VTE in our study was somewhat higher in the HF cohort (0.57%) than the COPD cohort (0.41%), which could reflect a higher prevalence in the HF cohort of mobility-limiting factors such as longer LOS, higher comorbidity burden, more extreme severity of illness, and more frequent ICU admissions.

Prior studies have reported mixed evidence on whether pharmacologic thromboprophylaxis improves in-hospital mortality.^31,61,64,65^ One explanation for this discrepancy is that different study populations presumably have different levels of risk for both VTE and major bleeding. In a recent real-world study of general medical inpatients from the PHD, inpatient thromboprophylaxis with enoxaparin was associated with significantly lower adjusted odds of PE-related mortality and overall mortality during index hospitalization, as compared with inpatient thromboprophylaxis with UFH.^61^ A study of PHD data on medical inpatients with obesity (a significant risk factor for VTE) reported similar findings.^65^ In our study, the adjusted odds of mortality during index hospitalization was approximately 10% lower with enoxaparin vs UFH thromboprophylaxis, a statistically significant and clinically meaningful difference. Rates of PE-related mortality also were consistently lower with enoxaparin in our study, but differences did not remain statistically significant after multivariable adjustment, probably because PE-related mortality was rare (the incidence was ≤0.2% in all 4 exposure groups). During the 90-day readmission period in our study, the adjusted odds of in-hospital mortality was slightly higher in the enoxaparin groups vs the UFH groups. Although the differences did not reach statistical significance, the trend differs from other measured clinical outcomes, which favored enoxaparin. The reason for this discrepancy is unclear but could indicate the presence of an unmeasured variable that slightly increased mortality risk in the enoxaparin groups after discharge. Given that the differences in 90-day mortality between exposure groups were small and not statistically significant, they would not be expected to have a significant impact on cost outcomes in our study.

Patients with COPD or HF not only are at heightened risk for VTE, but also can have comorbidities (such as liver disease and gastric ulcers) that independently increase bleeding risk.^66–69^ In addition, patients with COPD are at heightened risk for hemorrhagic stroke.^70,71^ Thus, it is crucial to weigh the risks and benefits of different anticoagulation strategies when considering thromboprophylaxis in these patient populations. In our study, patients with COPD or HF who received enoxaparin had significantly lower adjusted odds of bleeding during index hospitalization and during the 90 days after index discharge compared with patients who received UFH. Enoxaparin also was associated with lower bleeding risk compared with UFH in prior real-world PHD studies of general medical inpatients and medical inpatients with obesity.^61,65^ Both these studies and our study also identified lower rates of HIT with enoxaparin vs UFH.^61,65^ These findings are in line with safety data from two prior clinical trials comparing enoxaparin with UFH in medical inpatients and patients undergoing percutaneous coronary intervention.^49,72^ In contrast, the open-label PREVAIL study of patients with stroke unable to walk unassisted found that risk of major bleeding was higher with enoxaparin vs UFH.^47^ These findings highlight the need to carefully consider individual comorbidities and risks for VTE and bleeding when deciding on a thromboprophylactic agent, dose, and duration.

In our study, enoxaparin appears to be cost-effective compared with UFH. Among patients with COPD, adjusted mean total hospital costs per patient were 7.5% ($1280) lower during index hospitalization and 6.4% ($380) lower during the 90-day readmission period among enoxaparin recipients compared with UFH recipients. Similar patterns were observed in the HF cohort, where total mean hospital costs among enoxaparin recipients were $2677 lower during index hospitalization and $1024 lower during the 90-day readmission period, as compared with UFH recipients. Thromboprophylaxis with enoxaparin vs UFH also was associated with significant cost reductions in the prior PHD studies of general medical inpatients and medical inpatients with obesity.^61,65^ Moreover, decision analysis studies of medical inpatients also have demonstrated that thromboprophylaxis with enoxaparin was more cost-effective than UFH.^59,73^ Based on these findings, thromboprophylactic enoxaparin should be regarded as a cost-effective and potentially cost-saving strategy for preventing HA-VTE among appropriately selected medical inpatients, including patients with COPD or HF.

### Limitations

Most limitations of our study are intrinsic to retrospective studies of hospital administrative databases. Because patients were identified by ICD diagnosis codes, not by medical chart review, missing or erroneous codes or the use of insufficiently selective codes could have affected evaluations of study eligibility, covariates, and outcomes. For example, patients hospitalized for reasons other than acute COPD or HF exacerbation might have been underselected. We also were unable to stratify patients based on COPD or HF severity because the PHD lacks the relevant clinical data. In other studies, more severe HF and COPD were associated with higher risk of VTE and VTE-associated mortality.^15,74–76^ Events of PE also might have been underdetected, because PE can cause nonspecific signs and symptoms, such as dyspnea, that overlap with symptoms of HF and COPD exacerbation.^77,78^ In addition, because this is an observational study, no causal inference can be made between the exposure and outcome variables.

In any observational study, unmeasured factors can potentially confound relationships between exposures and outcomes of interest. The PHD lacks information on some VTE risk factors (such as smoking status) that could have contributed to unmeasured confounding. Of note, in our study, UFH recipients were significantly more likely to be admitted to the ICU and to have extreme severity of illness scores compared with patients who received enoxaparin. The reason for this is unclear, but it is noteworthy that renal disease was approximately twice as prevalent in the UFH groups as the enoxaparin groups. The absolute prevalence of renal disease was especially high among patients with HF (32% overall; 41% in UFH recipients). Heart failure causes venous congestion and reduces renal perfusion, ultimately leading to renal dysfunction.^79^ Unlike enoxaparin, UFH usually does not require dose adjustment in the setting of renal impairment (ie, creatinine clearance <30 mL/min), so UFH is often the anticoagulant of choice in the setting of HF.^80^ Although severe COPD can lead to right-sided HF,^81^ this appears to have less frequently necessitated the use of UFH in our study—only 31% of patients with COPD received UFH, compared with 42% of patients with HF. A final limitation of this study is that the PHD only tracks readmissions to the same hospital system, meaning that patients readmitted to other hospital systems would be lost to follow-up. However, loss to follow-up was unlikely to have differed between exposure groups.

## CONCLUSION

This study suggests that thromboprophylaxis with enoxaparin is associated with better outcomes and lower costs for preventing HA-VTE among medical inpatients with COPD or HF based on real-world evidence. When evaluating cost-effectiveness, it is vital to comprehensively evaluate healthcare costs, including those stemming from clinical outcomes and side effects of treatment. Quality and safety metrics of individual products should drive healthcare value measurement and healthcare decision making. Treatment decisions that enhance quality of care and the patient experience while reducing overall costs achieve value for both patients and healthcare systems. As new evidence-based data become available, clinicians and pharmacy and therapeutics committees should incorporate pharmacodynamic, pharmacokinetic, and health economics and outcomes research into decision making and protocols.

### Author Contributions

N.R., W.N., and A.A. had the idea for the study and designed the study. W.N. obtained funding for the study. N.R. and A.K. acquired the data. A.K. analyzed the data. A.A., N.R., K.S., and W.N. contributed to the writing of the article and reviewed it for important intellectual content. All authors contributed to the article and approved the submitted version.

### Disclosures

A.K. and N.R. are employees and shareholders of Premier Inc. K.S. and W.N. are employees and shareholders of Sanofi. A.A. has been a principal investigator or co-investigator of clinical trials sponsored by NIH/NIAID, NeuroRx Pharma, Pulmotect, Blade Therapeutics, Novartis, Takeda, Humanigen, Eli Lilly, PTC Therapeutics, OctaPharma, Fulcrum Therapeutics, and Alexion, and a speaker and/or consultant for Pfizer, Salix, Alexion, AstraZeneca, Bayer, Ferring, Seres, Spero, Eli Lilly, Nova Nordisk, Gilead, Renibus, GSK, Dexcom, HeartRite, and Aseptiscope; these relationships are unrelated to the current work.

## Supplementary Material

Online Supplementary Material
