# Supplementary material for: Effectiveness, Safety, and Costs of Thromboprophylaxis with Enoxaparin or Unfractionated Heparin Among Medical Inpatients With Chronic Obstructive Pulmonary Disease or Heart Failure
Source: J Health Econ Outcomes Res. 2024 Feb 20;11(1):44–56. doi: 10.36469/001c.92408 (PMC10883471; doi:10.36469/001c.92408)
Supplement: Online Supplementary Material [file jheor_2024_11_1_92408_194818.pdf]

Journal of Health Economics and Outcomes Research

Online Supplementary Material

Effectiveness, Safety, and Costs of Thromboprophylaxis with Enoxaparin or Unfractionated Heparin Among Medical Inpatients With Chronic Obstructive Pulmonary Disease or Heart Failure. *JHEOR*. 2024;11(1):??-??.
doi:10.36469/jheor.2024.92408

Table S1: Diagnostic Codes

This supplementary material has been provided by the authors to give readers additional information about their work.

This is an open-access article distributed under the terms of the Creative Commons Attribution 4.0 International License (CCBY-4.0). View this license's legal deed at http://creativecommons.org/licenses/by/4.0 and legal code at http://creativecommons.org/licenses/by/4.0/legalcode for more information.

Table S1. Diagnostic Codes

Diagnosis	Variable Type	Code Category	ICD-9-CM Code(s)	ICD-10-CM Code(s)
Chronic obstructive pulmonary disease	Cohort	Dx	490-496.9x, 500-505.9x, 506.4x	J40, J41.x, J42, J43.9, J44.x, J45.2x, J45.90x, J45.99x, J47.1, J47.9, J60, J61, J62.8, J63.x, J66.x, J64, J67.x, J68.4
Heart failure	Cohort	Dx	428-428.9x	I50.1, I50.20-I50.23, I50.33, I50.40-I50.43, I50.9
Deep vein thrombosis	Primary outcome measure	Dx	451.xx–453.xx	I80.xxx–I82.xxx
Pulmonary embolism	Primary outcome measure	Dx	415.1x	I26.9x and T80.xxxx–T82.xxxx
Major bleeding	Secondary outcome measure	Dx	430, 431, 432.x, 459.0, 578.x, 786.3x	I60.9–I62.9, R58, K92.0, K92.2, R04.x, D75.82
Heparin-induced thrombocytopenia	Secondary outcome measure	Dx	289.84	D75.82
Nephrotic syndrome	Comorbidity	Dx	581.xx	N04.x
Malignant hypertension	Comorbidity	Dx	401.0, 402-405.xx	I10, I11-I13.xx, I15.x
Myocardial infarction	Comorbidity	Dx	410-410.9x, 412-412.9x	I21.x, I22.x, I25.2, I23.x
Obesity	Comorbidity	Dx	278	E66
Fracture of lower limb	Comorbidity	Dx	820.x	S82.x
Inflammatory bowel disease	Comorbidity	Dx	555-556.x	K50-K51.xxx
Intubation	Comorbidity	Proc	I96.0x-96.5x, 96.6, 96.7x	09Hxxxx, 099xxxx, 0BHxxxx, 0B9xxxx, 0D9xxxx, 0T7xxxx, 0UHxxxx, 0WHxxxx, 0YQxxxx

Primary and secondary diagnoses and comorbidities were identified by the presence of a primary or secondary admission or discharge *International Classification of Diseases, Ninth Revision* (ICD)-9 or *Tenth Revision, Clinical Modification* (-10) code during the index hospitalization.
Note: All patients in the heart failure cohort had an ICD-CM code for heart failure; all patients in the COPD cohort had an ICD-CM code for COPD.
